# Inhibition of p38 Mitogen-Activated Protein Kinase Impairs Mayaro Virus Replication in Human Dermal Fibroblasts and HeLa Cells

**DOI:** 10.3390/v13061156

**Published:** 2021-06-17

**Authors:** Madelaine Sugasti-Salazar, Yessica Y. Llamas-González, Dalkiria Campos, José González-Santamaría

**Affiliations:** 1 Grupo de Biología Celular y Molecular de Arbovirus, Instituto Conmemorativo Gorgas de Estudios de la Salud, Panamá 0816-02593, Panama; msugasti@gorgas.gob.pa (M.S.-S.); qfb.y.llamas@gmail.com (Y.Y.L.-G.); dcampos@gorgas.gob.pa (D.C.); 2 Programa de Maestría en Microbiología Ambiental, Universidad de Panamá, Panamá 0824, Panama; 3 Programa de Doctorado en Ciencias Biológicas, Universidad de la República, Montevideo 11200, Uruguay

**Keywords:** Mayaro, MAPKs, p38, SB203580, PROTACs, NR-7, Losmapimod, replication, inhibition

## Abstract

Mayaro virus (MAYV) hijacks the host’s cell machinery to effectively replicate. The mitogen-activated protein kinases (MAPKs) p38, JNK, and ERK1/2 have emerged as crucial cellular factors implicated in different stages of the viral cycle. However, whether MAYV uses these MAPKs to competently replicate has not yet been determined. The aim of this study was to evaluate the impact of MAPK inhibition on MAYV replication using primary human dermal fibroblasts (HDFs) and HeLa cells. Viral yields in supernatants from MAYV-infected cells treated or untreated with inhibitors SB203580, SP600125, U0126, or Losmapimod were quantified using plaque assay. Additionally, viral protein expression was analyzed using immunoblot and immunofluorescence. Knockdown of p38⍺/p38β isoforms was performed in HDFs using the PROTACs molecule NR-7h. Our data demonstrated that HDFs are highly susceptible to MAYV infection. SB203580, a p38 inhibitor, reduced MAYV replication in a dose-dependent manner in both HDFs and HeLa cells. Additionally, SB203580 significantly decreased viral E1 protein expression. Similarly, knockdown or inhibition of p38⍺/p38β isoforms with NR-7h or Losmapimod, respectively, affected MAYV replication in a dose-dependent manner. Collectively, these findings suggest that p38 could play an important role in MAYV replication and could serve as a therapeutic target to control MAYV infection.

## 1. Introduction

Mayaro virus (MAYV) is an emerging, neglected arbovirus belonging to the *Togaviridae* family within the *Alphavirus* genus [1,2]. MAYV was isolated for the first time from the sera of forest workers in Trinidad and Tobago in 1954 [3]. Since then, several studies have revealed increasing activity of this virus in different Latin American countries, including Brazil, Peru, French Guiana, Ecuador, Bolivia, Colombia, Venezuela, Haiti, Panama, and Trinidad and Tobago [4,5,6,7,8,9,10,11,12,13,14].

MAYV causes the disease known as Mayaro fever, which is characterized by nonspecific symptoms, including fever, myalgia, rash, headache, retro-orbital pain, diarrhea, vomiting, lymphadenopathy, leukopenia, and arthralgia [15]. MAYV is transmitted through the bites of sylvatic mosquitos, mainly members of the *Haemagogus* genus [15]. However, several laboratory experiments suggest that urban mosquitos, such as *Aedes aegypti* or *Aedes albopictus*, can also spread this virus [16,17]. Consequently, there is a growing concern about MAYV’s potential to cause a larger-scale epidemic similar to other arboviruses, such as chikungunya and Zika [18,19].

As an obligatory intracellular pathogen, MAYV relies on the cell machinery to efficiently replicate. In line with this, Barroso and colleagues observed that MAYV activates casein kinase 2 in an early stage of infection in Vero cells [20]. The inhibition of casein kinase 2 with dichloro-1-(beta-D-ribofuranosyl) benzimidazole decreased MAYV-induced cytopathic effects [20]. Shortly after, the same research group found that MAYV was able to modify glucose metabolism through activation of the enzyme 6-phosphofructo 1-kinase [21]. Additionally, our laboratory showed that proteasome inhibitors MG132 and Lactacystin decrease MAYV replication in a dose-dependent manner, suggesting that the ubiquitin-proteasome system plays an essential role in MAYV replication [22].

In the last decade, growing evidence has suggested that kinases are critical cellular factors for replication in a range of RNA and DNA viruses, and it has been proposed that chemical inhibition of these proteins may represent an antiviral therapeutic strategy [23,24]. Mitogen-activated protein kinases (MAPKs) control signal transduction pathways that regulate essential cell activities, including proliferation, growth, differentiation, cytokine production, motility, stress response, survival, and apoptosis [25]. Classic MAPKs include p38, c-Jun N-terminal kinase (JNK), and extracellular signal-regulated kinases (ERK1/2) [25]. These enzymes are serine/threonine kinases that are activated by a variety of extracellular stimuli; after activation, the signal is then propagated through the phosphorylation of diverse substrates. Among these substrates are transcription factors, which modulate specific gene expression programs, allowing cells to adapt to different physiological conditions [25]. Previous studies have demonstrated that viruses from various families are able to exploit MAPK activity to favor their replication [26,27,28,29]. However, whether MAYV is capable of manipulating these pathways to competently replicate has not yet been investigated. The aim of this study was to assess the effect of MAPK inhibition on MAYV replication using in vitro cell models of infection and an array of small inhibitory molecules.

## 2. Materials and Methods

### 2.1. Cell lines and Reagents

Normal human dermal fibroblasts (HDFs) from adults (PCS-201-012), Vero-E6 (CRL-1586) (both obtained from the American Type Culture Collection, Manassas, VA, USA) and HeLa cells (kindly provided by Dr. Carmen Rivas, CIMUS, Coruña, Spain) were grown in Dulbecco’s Modified Eagle’s Medium (DMEM) (for HDFs) or Minimal Essential Medium (MEM) (for Vero-E6 and HeLa cells). Both media were supplemented with 10% heat-inactivated fetal bovine serum (FBS), 2 mM of _L_-Glutamine, and 1% penicillin-streptomycin antibiotic solution (all culture reagents were obtained from Gibco, Waltham, MA, USA). All cell lines were incubated at 37 °C under a 5% CO_2_ atmosphere. The MAPK inhibitory compounds SB203580 (p38), SP600125 (JNK) and U0126 (MEK1/2) were obtained from Cell Signaling Technology (Danvers, MA, USA). NR-7h, a p38α and p38β isoform degrader, was acquired from Tocris (Minneapolis, MN, USA), and Losmapimod (p38 inhibitor under clinical evaluation) was obtained from Selleckchem (Houston, TX. USA). All compounds were dissolved in Dimethyl sulfoxide (DMSO, Sigma-Aldrich, Saint Louis, MI, USA) at 10 mM concentration and stored at −20 °C until use. Working solutions of each compound were prepared in DMEM or MEM at the indicated concentrations.

### 2.2. Virus Strain and Propagation

A Mayaro strain (MAYV, AVR0565, San Martin, Peru) [30] was acquired from the World Reference Center for Emerging Viruses and Arboviruses (WRCEVA) at the University of Texas Medical Branch (UTMB, Galveston, TX, USA) and kindly provided by Dr. Scott Weaver. The MAYV strain was propagated, titrated, aliquoted, and stored as previously reported [22].

### 2.3. Cell Toxicity Analysis of Small Inhibitory Molecules

The cytotoxicity of inhibitors was evaluated using the MTT method as previously described [22]. Approximately 2.5 × 10^4^ HDFs or HeLa cells were seeded in 96-well plates in DMEM or MEM without phenol red. The next day, cell lines were treated with increasing concentrations of SB203580 (0.5, 2, and 10 µM), SP600125 (0.5, 1, and 5 µM), U0126 (1, 5, and 10 µM), NR-7h (0.05, 0.2, and 1 µM), Losmapimod (1, 5, and 10 µM), or DMSO (0.1%), which served as control, and incubated for 24 h. Then, 5 mg/mL of 3-(4,5-Dimethyl-2-thiazolyl)-2,5-diphenyltetrazolium bromide (MTT, Sigma-Aldrich, Saint Louis, MI, USA) solution in PBS was added to the cells and incubated for an additional 4 h. Formazan crystals were dissolved in a solution of 4 mM HCL and 10% Triton X-100 in isopropanol, and absorbance was measured at 570 nm using a microplate reader spectrophotometer (BioTek, Winooski, VT, USA). Results are shown as the percentage of viable cells relative to untreated control cells.

### 2.4. Viral Infection Assay

HDFs or HeLa cells grown in 12- or 24-well plates were infected with MAYV at a multiplicity of infection (MOI) of 1 and 10, respectively. In all assays, the cells were pre-treated with DMSO (as a control) or with each inhibitor tested at the indicated concentrations and times. Cell supernatants were collected, and viral titers for each experimental condition were quantified using plaque assay. To analyze the E1 viral protein, cell lysates were obtained from mock- or MAYV-infected HDFs or HeLa cells treated or untreated with the inhibitors. Finally, for the virucidal experiments, 10^6^ PFU of MAYV were incubated in serum-free MEM with DMSO or MAPK inhibitors SB203580 (10 µM), SP600125 (1 µM), or U0126 (10 µM) for 1 h at 37 °C. After this, the viral particles were measured using plaque assay.

### 2.5. Virus Titration Assay

Virus titers in cell supernatants were quantified using plaque assay following the procedure described in a previous work [22]. Confluent Vero-E6 cells grown in 6-well plates were infected with 10-fold serial dilutions from cell supernatants for 1 h at 37 °C. After virus absorption, the inoculum was removed and the cells were overlaid with a solution of agar (1%) in MEM supplemented with 2% FBS, and incubated for 3 days at 37 °C. After this, the agar was eliminated and the cells were fixed with 4% formaldehyde solution in PBS and stained with 2% crystal violet prepared in 30% methanol. Lastly, the number of plaques was counted, and the viral titers were recorded as plaque-forming units per milliliter (PFU/mL).

### 2.6. Analysis of mRNA Expression by Quantitative RT-PCR

Total RNA was extracted from MAYV-infected HDFs using an RNeasy kit (Qiagen, Valencia, CA, USA) following the manufacturer’s instructions. cDNA was synthesized from 1 µg of RNA using a High-Capacity cDNA Reverse Transcription kit, and quantitative RT-PCR was performed using Power SYBR Green PCR Master Mix in a QuantStudio^TM^ 5 thermocycler (all from Applied Biosystems, Foster City, CA, USA) to detect the following immune response genes: *MDA5*, *ISG15*, *MxA*, *RIG-I*, *OAS2*, *AIM2*, *IL1-β*, *TNF-α*, *IL-6*, *RANTES*, *IL-8*, *TLR3*, *TLR7*, *IRF3*, *IRF7*, *IFN-α*, and *IFN-β*. Specific primers for the mRNA expression analysis are listed in Table S1 (Supplementary Material). Relative mRNA expression was determined using the *β-actin* gene for normalization according to the ∆∆ CT method [31].

### 2.7. Protein Analysis

Viral and cellular protein levels were assessed using immunoblot as previously performed [22]. Protein extracts were obtained from mock- or MAYV-infected HDFs or HeLa cells that were treated with different inhibitors or a control in Laemmli buffer with 10% Dithiothreitol (Bio-Rad, Hercules, CA, USA). Proteins were fractionated in SDS-PAGE, transferred to nitrocellulose membranes, and blocked in a 5% non-fat solution in T-TBS buffer for 30 min. Then, membranes were incubated with the following primary antibodies: rabbit polyclonal anti-E1 (validated in our laboratory [22]), rabbit monoclonal anti-GAPDH (D16H11, Cat. # 5174), rabbit monoclonal anti-β-actin (D6A8, Cat. # 8457), and rabbit monoclonal anti-p38 MAPK (D13E1, Cat. # 8690) (all from Cell Signaling Technology, Danvers, MA, USA). After this, the membranes were washed 3 times in T-TBS buffer and incubated with HRP-conjugated goat anti-rabbit secondary antibody (Cat. # 926-80011, LI-COR, Lincoln, NE, USA) at room temperature for 1 h. Finally, the membranes were incubated with SignalFire^TM^ ECL Reagent (Cell Signaling Technology, Danvers, MA, USA) for 5 min and the quimioluminescent signal was detected with a C-Digit scanner (LI-COR, Lincoln, NE, USA).

### 2.8. Knockdown of p38α and p38β Isoforms Using Proteolysis Targeting Chimeras (PROTACs)

HDFs grown in 12-well plates were treated with increasing concentrations of the PROTACs compound NR-7h or DMSO, and at the indicated times, p38α/β protein levels in the cell lysates were evaluated using immunoblot.

### 2.9. Immunofluorescence Analysis

Immunofluorescence experiments were performed as previously reported [22]. Briefly, HDFs grown on glass coverslips were infected with MAYV at an MOI of 1 and treated with the inhibitors or a control. After 24 h of infection, cells were fixed, permeabilized, blocked, and stained overnight at 4 °C with an anti-MAYV mouse ascitic fluid (kindly provided by Dr. Scott Weaver, WRCEVA-UTMB, Galveston, TX, USA). Next, cells were incubated with a goat anti-mouse secondary antibody (Alexa Flour 488 or Alexa Flour 568, Invitrogen, Carlsbad, CA, USA) for 1 h in the dark. Lastly, coverslips were mounted on slides with Prolong Diamond Antifade Mountant with Dapi (Invitrogen, Carlsbad, CA, USA), and images were acquired with an FV1000 Flowview confocal microscope (Olympus, Lombard, IL, USA). The images were analyzed with ImageJ software.

### 2.10. Data Analysis

Experimental data were analyzed using the Mann–Whitney test and the One- or Two-way ANOVA test, followed by Dunnett’s or Sidak’s post-test. GraphPad Prism software version 9.1.0 for Mac was used to perform all statistical tests and create the graphics. A *p* value < 0.05 was considered statistically significant. All experiments were performed at least 3 times with 3 replicates. For each experiment, the mean and standard deviation are shown.

## 3. Results

### 3.1. MAYV Infects Primary Human Dermal Fibroblasts in a Time-Dependent Manner

**Figure 1:**
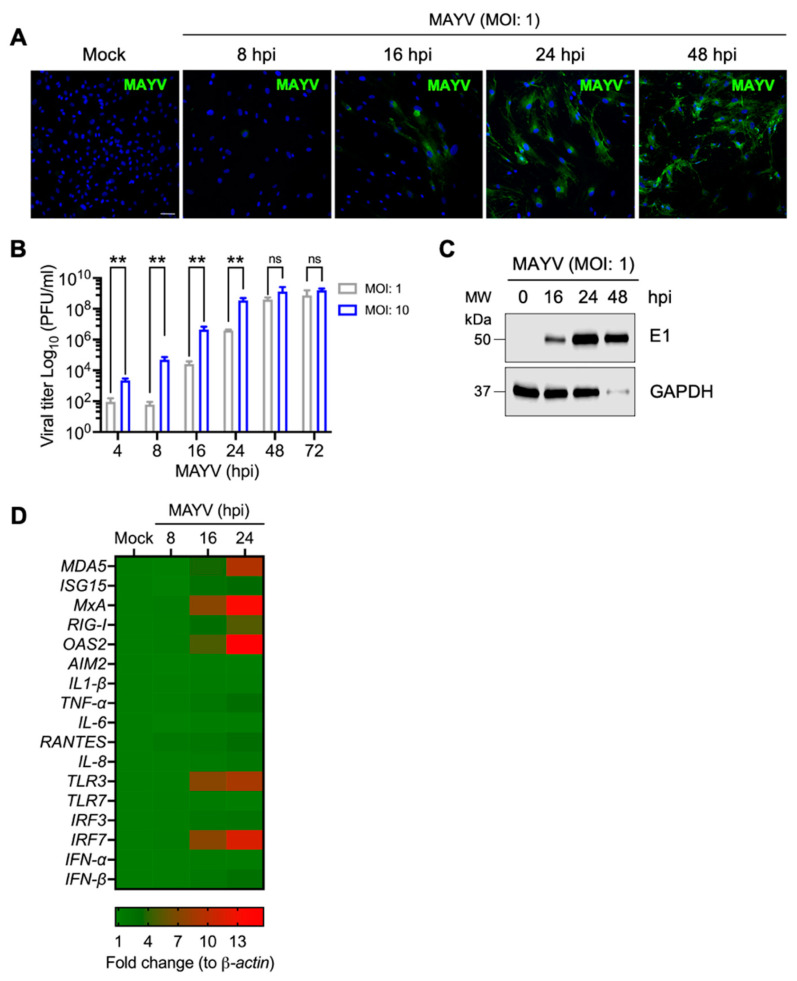
Primary human dermal fibroblasts are susceptible to MAYV infection. (**A**) HDFs grown on glass coverslips were infected with MAYV at an MOI of 1; at different time points, the cells were stained with an anti-MAYV mouse primary antibody, followed by an Alexa-Flour 488 mouse secondary antibody. Cell nuclei were stained with Dapi. Images were captured with a confocal microscope and analyzed with ImageJ software. Scale bar, 50 µm. (**B**) HDFs were infected with MAYV at the indicated MOI, and viral progeny production in cell supernatants collected at different time intervals was measured using plaque assay. Viral titers are represented as plaque-forming units per milliliter (PFU/mL). Statistically significant differences were evaluated with the Mann–Whitney test: ** *p* < 0.01; ns: non-significant. (**C**) The levels of viral E1 protein at different time points were determined in cell lysates from mock- or MAYV-infected HDFs using immunoblot. GAPDH protein was used as a loading control. MW: molecular weight. kDa: kilodaltons. (**D**) Heat map showing the expression profile of specific interferon-stimulated genes, inflammatory cytokines, chemokines, and transcription factor genes implicated in the immune response in mock- or MAYV-infected HDFs using quantitative RT-PCR.

In order to assess whether primary HDFs are susceptible to MAYV infection, we performed an infection kinetics experiment in HDFs with MAYV at an MOI of 1 and evaluated the cytopathic effect after viral infection. In this experiment, we noted a slight cytopathic effect at 24 h post-infection (hpi), which significantly increased at 48 and 72 hpi (Figure S1). To determine whether the cytopathic effect observed was associated with an increase in the number of MAYV-infected cells, we decided to study the presence of MAYV antigens in infected HDFs at different times using an immunofluorescence confocal microscope. As expected, this analysis revealed a time-dependent increase in the number of MAYV-positive cells (Figure 1A). To quantify the viral particles present in the cell supernatants, we completed a similar experiment in which HDFs were infected with MAYV at an MOI of 1 or 10; at different times post-infection, we measured the viral progeny yields using plaque assay. As shown in Figure 1B, we detected viral particles in the supernatants of MAYV-infected HDFs as early as 4 hpi, and there was a clear time-dependent rise in viral titers for both MOIs tested (Figure 1B). To confirm these results, we evaluated the expression of structural E1 viral protein in cell lysates obtained from MAYV-infected HDFs using immunoblot. In this assay, we detected E1 at 16 hpi, and this protein increased over time (Figure 1C). Finally, we investigated if infecting HDFs with MAYV elicits the expression of immune response genes. To accomplish this, we extracted total RNA from mock- or MAYV-infected cells and then used quantitative RT-PCR to measure the expression levels of specific interferon-stimulated genes, inflammatory cytokines, chemokines, and transcription factor genes implicated in the immune response. As shown in Figure 1D, we observed a strong expression of the *MDA5*, *MxA*, *RIG-I*, *OAS2*, *TLR3*, and *IRF7* genes, while the *ISG15*, *TNF-α*, *RANTES*, *IRF3*, and *IFN-β* genes were expressed to a lesser extent (Figure 1D). Taken together, the described results indicate that MAYV efficiently infects HDFs and stimulates the expression of specific immune response genes.

### 3.2. p38 Inhibitor SB203580 Diminishes MAYV Progeny Production in HDFs and HeLa Cells

**Figure 2:**
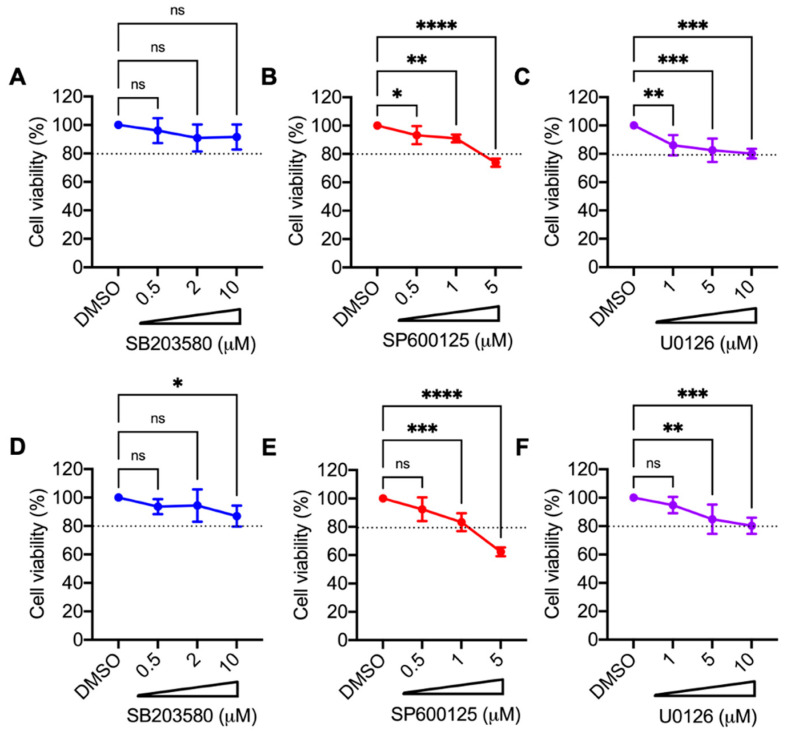
Toxicity of MAPK inhibitors SB203580, SP600125, and U0126 in HDFs or HeLa cells. HDFs (**A**–**C**) or HeLa (**D**–**F**) cells were treated with the indicated concentrations of each MAPK inhibitor, or DMSO (0.1%) as a control, and after 24 h of incubation, cell viability was assessed using the MTT method. Statistically significant differences were assessed with the One-way ANOVA test followed by Dunnett’s post-test: * *p* < 0.05; ** *p* < 0.01; *** *p* < 0.001; **** *p* < 0.0001; ns: non-significant. The dotted line on the graphs indicates a cell viability of 80%.

**Figure 3:**
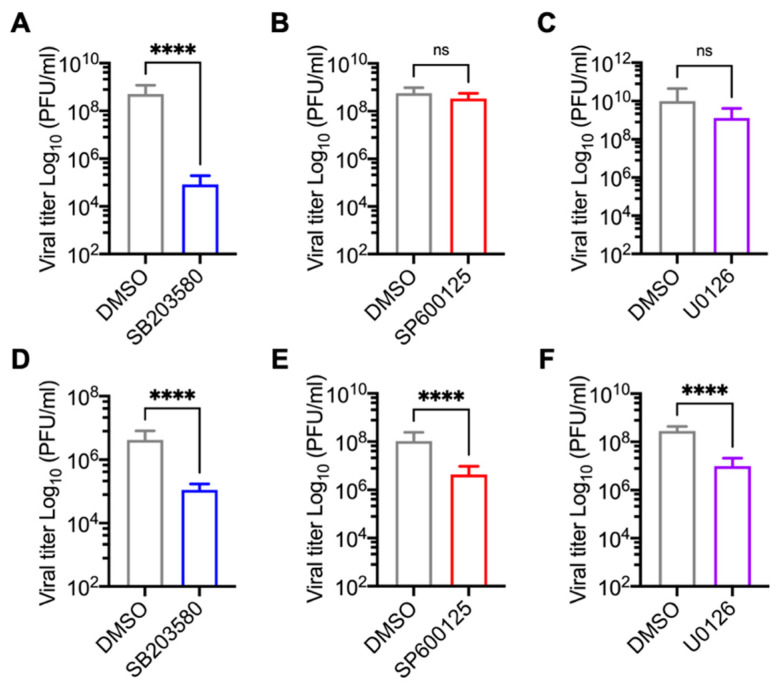
Inhibition of p38 with SB203580 decreases MAYV progeny production in HDFs and HeLa cells. HDFs (**A**–**C**) or HeLa (**D**–**F**) were pre-treated with SB203580, SP600125, or U0126 at concentrations of 10, 1, and 10 µM, respectively, for 1 h. Then, the compounds were removed and the cells were infected with MAYV at an MOI of 1 (HDFs) or 10 (HeLa cells). After virus adsorption, the MAPK inhibitors dissolved in fresh medium were added to the cells and incubated for 24 h. Viral progeny production in cell supernatants from each experimental condition was quantified using plaque assay. Statistically significant differences were analyzed with the Mann–Whitney test: **** *p* < 0.0001 and ns: non-significant.

To explore the potential role of the MAPKs p38 and JNK as well as the ERK1/2 cellular pathways in MAYV replication, we used a pharmacological approach. Our experiments employed small inhibitory molecules that block the activity of these protein kinases and ultimately affect the downstream cellular signaling events controlled by them. Specifically, we selected SB203580, a class of pyridinyl imidazole which is a well-characterized inhibitor of p38α and p38β isoforms [32]; SP600125, an anthrapyrazolone molecule with a potent inhibitory effect on JNK1, -2 and 3- isoforms [33]; U0126, which is a selective noncompetitive inhibitor of MEK1 and MEK2, the kinases responsible for phosphorylation and activation of the ERK1/2 pathway [34]. In order to examine the toxicity of the MAPK inhibitors SB203580, SP600125, and U0126 in primary HDFs and HeLa cells, we treated both cell lines with increasing concentrations of each inhibitor and then determined cell viability using the MTT method. As shown in Figure 2, in the case of the SB203580 and U0126 inhibitors, we observed a cell viability of 80% or higher in both cell lines at the maximum dose tested (10 µM) (Figure 2, panels A and C–F). Conversely, in both cell lines treated with the highest dose of the SP600125 inhibitor (5 µM), cell viability was less than 80% (Figure 2, panels B,E). These results indicate that the p38 and MEK kinase inhibitor concentrations used were well-tolerated in the cell lines tested, whereas the JNK inhibitor showed cell toxicity. Therefore, we used SP600125 at a concentration of 1 µM in the subsequent experiments. To evaluate the impact of the MAPK inhibitors on MAYV, we used two human cell lines as infection models: HDFs, which are normal primary cells, and HeLa cells, which are cancer cells derived from a cervix tumor and have previously been shown to be susceptible to MAYV infection [22]. We pre-treated the HDFs or HeLa cells with the inhibitors or DMSO at the indicated concentrations for 1 h. Then, we removed the compounds and infected the HDFs and HeLa cells with MAYV at an MOI of 1 and 10, respectively. After 1 h of virus absorption, we added the MAPK inhibitors or DMSO in fresh medium. After a 24-h incubation period, we collected the cell supernatants to quantify viral production using a plaque assay. In this experiment, when compared to DMSO-treated cells, HDFs treated with the p38 inhibitor SB203580 showed a significant reduction in viral titers, while a less pronounced reduction was observed in treated HeLa cells (Figure 3A,D). In the case of the JNK and MEK inhibitors, SP600125 and U0126, respectively, we noted a minor but significant decrease in viral progeny production in the tumoral HeLa cells only (Figure 3B,C,E,F). To verify whether the observed effect of the MAPK inhibitors was due directly to MAYV infectivity, we carried out a virucidal activity assay. For this, we added 10^6^ PFU of MAYV in MEM medium along with each inhibitor or DMSO (control), incubated the solutions for 1 h at 37 °C, and then directly quantified the remaining virus under each experimental condition as previously indicated. As shown in Figure S2, we did not find a decrease in viral titers in the solutions containing MAYV and the MAPK inhibitors, indicating that these compounds do not have direct virucidal activity. Taken together, these findings suggest that the p38 inhibitor SB203580 significantly decreases MAYV particles’ production for both of the cell types tested, whereas the observed effects of the JNK and MEK inhibitors on MAYV production are cell type-dependent.

### 3.3. Decrease in MAYV Replication in HDFs and HeLa Cells Treated with p38 Inhibitor SB203580 Is Dose-Dependent

**Figure 4:**
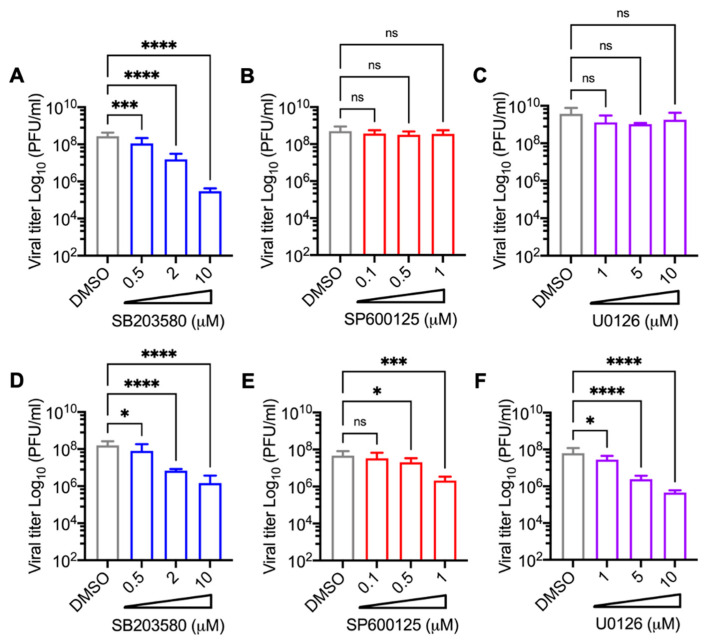
MAPK p38 inhibitor SB203580 reduces MAYV titers in HDFs and HeLa cells in a dose-dependent manner. HDFs (**A**–**C**) or HeLa (**D**–**F**) cells were pre-treated with increasing concentrations of SB203580, SP600125, or U0126 and then infected with MAYV as previously performed. After 24 h of infection, MAYV titers were measured in cell supernatants using plaque assay. Statistically significant differences were evaluated with the One-way ANOVA test followed by Dunnett’s post-test: * *p* < 0.05; *** *p* < 0.001; **** *p* < 0.0001 and ns: non-significant.

To investigate whether the effect of the MAPK inhibitors on MAYV replication was affected by the drug concentration, we performed a dose-response experiment. HDFs or HeLa cells were pre-treated with the MAPK inhibitors and then infected with MAYV as previously indicated. Then, we quantified the viral titer production in the cell supernatants after 24 h of incubation with the inhibitors or DMSO as previously described. This analysis revealed that treatment with the p38 inhibitor SB203580 led to a significant dose-dependent decrease in viral titers in both cell lines tested (Figure 4A,D). On the other hand, following treatment with the JNK and MEK inhibitors, SP600125 and U0126, respectively, a dose-dependent decrease in viral replication was only observed in the HeLa cells (Figure 4B,C,E,F). These results confirm that the p38 inhibitor SB203580 demonstrates dose-dependent inhibition of MAYV replication regardless of the cell type tested.

### 3.4. SB203580 Affects the Expression of MAYV Structural E1 Protein in Both HDFs and HeLa Cells

**Figure 5:**
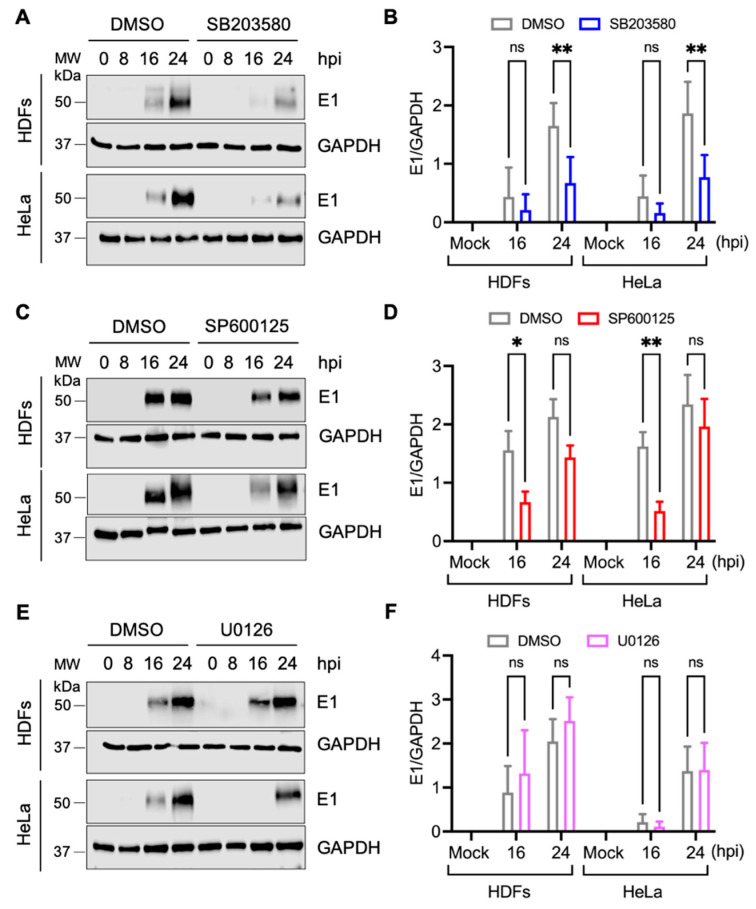
SB203580 disturbs MAYV structural E1 protein expression in both HDFs and HeLa cells. HDFs or HeLa cells were pre-treated with SB203580 (**A**), SP600125 (**C**), or U0126 (**E**) and then infected with MAYV as previously indicated. At different times post-infection, structural E1 viral protein levels were evaluated using immunoblot. GAPDH protein was used as a loading control. MW: molecular weight. kDa: kilodaltons. (**B**,**D**,**F**) Densitometric analysis of E1 protein was performed using ImageJ software and normalized with GAPDH protein. Data were analyzed with the Two-way ANOVA test followed by Sidak’s post-test. Statistically significant differences are shown: * *p* < 0.05; ** *p* < 0.01 and ns: non-significant.

Previous studies have revealed that inhibiting MAPK pathways can affect the expression of several viral proteins [28,35,36]. Thus, to explore a possible mechanism by which MAPK inhibitors affect MAYV replication, we used immunoblot to evaluate the structural E1 protein levels in cell lysates from MAYV-infected HDFs or HeLa cells treated with DMSO or the inhibitors SB203580, SP600125, or U0126 at different times post-infection. As shown in Figure 5, inhibiting p38 with SB203580 promoted a significant reduction in viral E1 protein levels after 24 h of infection when compared to DMSO-treated cells in both of the cell lines assessed (Figure 5A,B). In the case of SP600125, we observed a slight decrease in E1 in both HDFs and HeLa cells after only 16 h of infection (Figure 5C,D). In contrast, we did not observe any differences in E1 levels in the cells treated with U0126 (Figure 5E,F). These results suggest that inhibiting p38 effectively disrupts the expression of MAYV’s E1 protein independent of the cell type examined.

### 3.5. Knockdown of p38α and p38β Isoforms in HDFs with the PROTACs Molecule NR-7h Reduces MAYV Replication

**Figure 6:**
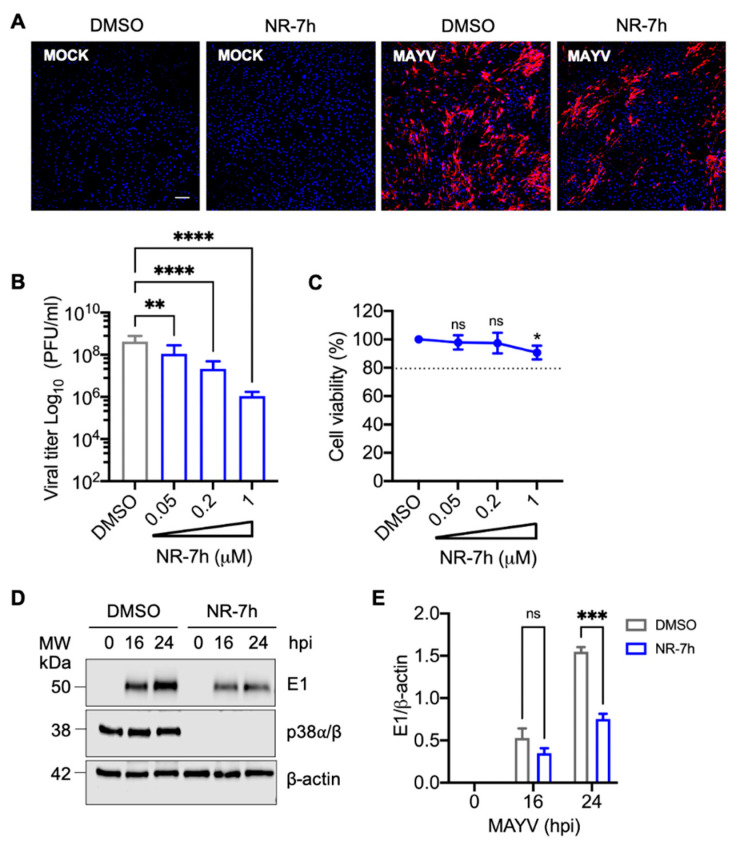
Chemically-induced degradation of p38α and p38β isoforms using the PROTACs compound NR-7h affects MAYV replication. (**A**) HDFs were pre-treated with DMSO or NR-7h at 1 µM for 3 h and then the cells were infected with MAYV as previously indicated. After 24 h of infection, MAYV antigen-positive cells were analyzed using an immunofluorescence assay as previously stated. Cell nuclei were stained with Dapi. Images were obtained with a confocal microscope and analyzed with ImageJ software. Scale bar, 100 µm. (**B**) HDFs were pre-treated with increasing concentrations of NR-7h and then infected with MAYV as previously performed. After 24 h of infection, viral progeny production in cell supernatants was assessed using plaque assay. Viral titers were expressed as plaque-forming units per milliliter (PFU/mL). (**C**) HDFs were treated with the indicated concentrations of NR-7h for 24 h and then cell viability was estimated as previously performed. The dotted line on the graph indicates a cell viability of 80%. (**D**) HDFs were pre-treated with NR-7h and then infected as stated above. At several time points, viral E1 or p38α/p38β protein levels were analyzed using immunoblot. Β-actin protein was used as a loading control. MW: molecular weight. kDa: kilodaltons. (**E**) Intensity bands of viral E1 protein were analyzed with ImageJ software and normalized with Β-actin protein. Data were analyzed with the One- or Two-way ANOVA test followed by Dunnett’s or Sidak’s post-test. Statistically significant differences are denoted: * *p* < 0.05; ** *p* < 0.01; *** *p* < 0.001; **** *p* < 0.0001 and ns: non-significant.

Our previous results with SB203580 indicated that p38 kinase may play a key role in MAYV replication. In order to verify this hypothesis, we used a new experimental pharmacological approach known as Proteolysis Targeting Chimeras (PROTACs) for the following experiments [37]. PROTACs have emerged as a promising therapeutical tool that allow proteins to be targeted for degradation using a ubiquitin proteasome system-dependent mechanism [37]. These small hetero-bifunctional molecules contain three vital structural elements: a protein of interest ligand, an E3 ubiquitin ligase ligand, and a linker that joins these two components [38]. PROTACs function by recognizing a specific protein substrate and recruiting an E3 ubiquitin ligase that promotes the polyubiquitination of the protein of interest and its subsequent degradation in the proteasome [37,38]. The p38 MAPK family includes four isoforms: p38α, p38β, p38γ, and p38δ [39,40]. While p38α and p38β are ubiquitously expressed in most tissues, p38γ and p38δ have restricted expression [40]. Recently, it has been reported that nanomolar concentrations of the PROTACs compound NR-7h are able to induce the specific degradation of p38α and p38β isoforms, thereby blocking stress- and cytokine-induced downstream signaling by p38 [41]. With this in mind, we tested the effect of NR-7h in our cell infection model. For this purpose, we chose primary HDFs and treated them with increasing concentrations of NR-7h for 24 h; then, we evaluated the p38α and p38β levels using an antibody that recognizes both isoforms. In this analysis, we found high levels of p38α/p38β in HDFs treated with DMSO (Figure S3A). However, we did not detect p38 in the cells treated with NR-7h from concentrations as low as 50 nM (Figure S3A,C). In order to determine the time at which p38α/p38β degradation occurs, we performed a kinetic experiment in which HDFs were treated with NR-7h at a 1 µM concentration at different intervals, and the p38 protein levels were assessed. As shown in Figure S3 (panels B and D), after 2 h of NR-7h treatment, we observed that more than 95% of p38α/p38β had been degraded. These results indicate that NR-7h quickly and effectively induces p38α/p38β degradation in HDFs. To gain insights about the effects of NR-7h-induced p38α/p38β degradation on MAYV replication, we pre-treated HDFs with NR-7h or DMSO for 3 h and then infected the cells with MAYV in the presence of NR-7h or DMSO. After 1 h of virus absorption, we removed the inoculum, added NR-7h or DMSO in fresh medium, and then incubated the samples for 24 h. After this, we analyzed the presence of MAYV antigens using immunofluorescence. In DMSO-treated cells, we observed a high number of MAYV antigen-positive cells (Figure 6A). In contrast, we found a clear reduction in MAYV antigen-positive cells in NR-7h-treated cells (Figure 6A). To confirm the preceding results, we pre-treated HDFs with different concentrations of NR-7h and then infected the cells with MAYV as previously indicated. After 24 h of incubation, we collected the cell supernatants and quantified the viral particles’ production using plaque assay. In this experiment, we observed a dose-dependent decrease in MAYV viral titers in the cells treated with NR-7h (Figure 6B). This NR-7h-induced decrease in MAYV viral titers did not appear to be associated with cell toxicity, as revealed by our cell viability analysis (Figure 6C). Given that inhibiting p38 with SB203580 disrupted the expression of the MAYV structural E1 protein, we wanted to explore the effect of p38α/p38β degradation on viral E1 protein expression. Accordingly, we performed an infection kinetics experiment in which HDFs were infected with MAYV and treated with DMSO (control) or NR-7h as previously indicated; viral E1 protein levels were assessed using immunoblot at different time points. This assay revealed that p38α/p38β knockdown promoted a decrease in viral E1 protein levels at 24 hpi (Figure 6D,E). Together, these findings confirm that p38 could play an important role in MAYV replication.

### 3.6. Losmapimod, a p38 Inhibitor under Clinical Evaluation, Decreases MAYV Replication in HDFs

**Figure 7:**
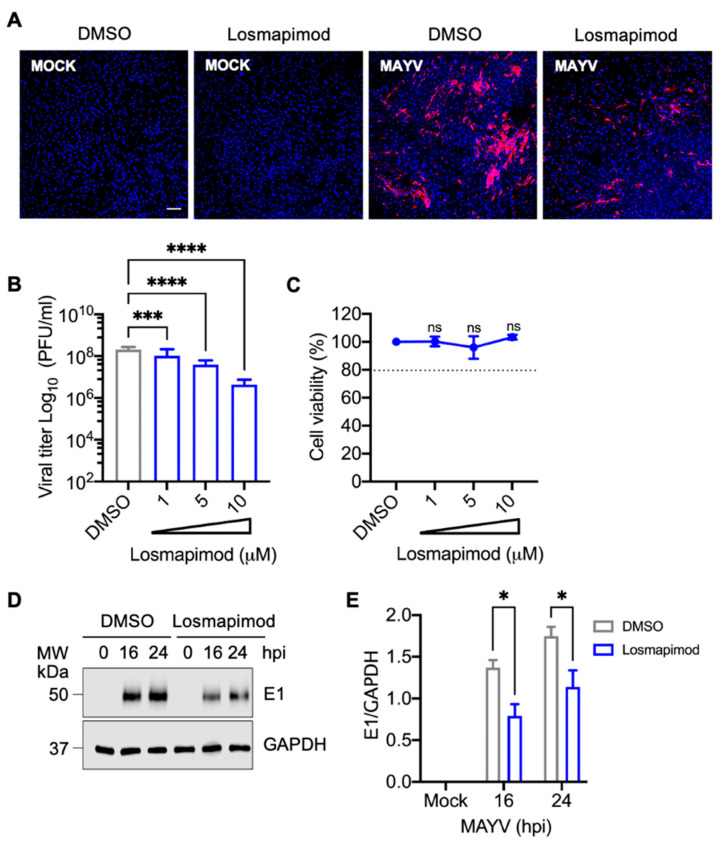
Losmapimod treatment of HDFs reduces MAYV replication in a dose-dependent manner. (**A**) HDFs grown on glass coverslips were pre-treated with Losmapimod at a concentration of 10 µM or DMSO for 1 h. After this, cells were infected with MAYV and incubated with DMSO or Losmapimod in fresh medium for 24 h. Then, MAYV antigen-positive cells were evaluated as previously stated. Cell nuclei were stained with Dapi. Images were captured with a confocal microscope and examined with ImageJ software. Scale bar, 100 µm. (**B**) HDFs were pre-treated with Losmapimod at the indicated concentrations and then infected as previously stated. Viral progeny production in cell supernatants collected at 24 hpi was analyzed using plaque assay. Viral titers were expressed as plaque-forming units per milliliter (PFU/mL). (**C**) HDFs were treated with the indicated concentrations of Losmapimod and after 24 h of incubation, cell viability was assessed as previously indicated. The dotted line on the graph indicates a cell viability of 80%. (**D**) Viral E1 protein levels were analyzed in Losmapimod-treated and untreated HDFs using immunoblot. GAPDH protein was used as a loading control. (**E**) Densitometric analysis of E1 viral protein was performed with ImageJ software and normalized with GAPDH protein. Data were analyzed with the One- or Two-way ANOVA test followed by Dunnett’s or Sidak’s post-test. The statistically significant differences are shown: * *p* < 0.05; *** *p* < 0.001; **** *p* < 0.0001 and ns: non-significant.

Since p38 has been implicated in different pathologies, including inflammatory diseases, neurological disorders, and cancer, pharmaceutical companies have developed diverse small inhibitory molecules targeting this kinase [39,40,42,43]. Among them, Losmapimod is a highly selective and potent inhibitor against p38α and p38β isoforms that has been evaluated in different clinical trials and shown to be well-tolerated and safe [44,45,46]. Therefore, we wanted to investigate the potential antiviral activity of Losmapimod with MAYV. For this, we pre-treated HDFs with Losmapimod or DMSO, infected the cells as previously performed, and assessed the number of MAYV antigen-positive cells using an immunofluorescence assay. As shown in Figure 7, we observed a reduction in MAYV antigen-positive cells with Losmapimod treatment compared to DMSO-treated cells (Figure 7A). To determine the impact of this drug on viral progeny production, we pre-treated HDFs with increasing concentrations of Losmapimod, infected the cells as previously indicated, and incubated them with Losmapimod or DMSO for 24 h. After this, we collected the cell supernatants and quantified the viral progeny production as previously performed. In this experiment, we found that Losmapimod-treated cells showed a concentration-dependent decrease in MAYV titers (Figure 7B), and this effect was not due to the cell toxicity of the inhibitor (Figure 7C). Finally, we analyzed E1 protein levels in HDFs treated with DMSO or Losmapimod at different time points after infection. This analysis revealed that Losmapimod induces a reduction in viral E1 protein expression. These results confirm that p38 plays a relevant role in MAYV replication and also suggest that this kinase could represent a pharmacological target to control MAYV infection.

## 4. Discussion

MAYV is an emerging but poorly characterized arbovirus with increasing recorded activity in the Latin American region [9]. Despite its potential to cause an epidemic, there are no approved vaccines or antiviral compounds to combat this pathogen [1]. As a result, it is imperative to find molecules with anti-MAYV activity. MAYV, like other viruses, must manipulate an array of the host’s cellular process in order to successfully replicate [20,21,22]. Therefore, a reasonable strategy for discovering possible treatments consists of identifying key cellular factors or pathways required for MAYV replication. The MAPKs p38, JNK, and ERK1/2 are enzymes that regulate essential cell functions and have emerged as crucial cellular factors implicated in different stages of the viral cycle. Moreover, various authors have proposed that inhibiting these enzymes could represent a therapeutic antiviral approach [23,28,35,47].

In this study, we analyzed the impact of inhibiting the p38, JNK, and ERK1/2 MAPK pathways on MAYV replication using two cell models of infection: primary HDFs or HeLa cells. Previously, we have shown the utility of HeLa cells as a MAYV infection model to identify potential antiviral drugs [22,48]. However, we did not know if HDFs were susceptible to this virus. For this reason, we first performed an infection kinetics experiment with these cells; at different time points we evaluated various parameters, among them: cytopathic effects, the presence of MAYV proteins in infected cells, and viral progeny production in cell supernatants. Our results demonstrate that HDFs are highly susceptible to MAYV infection. Moreover, MAYV is able to elicit the expression of specific interferon-stimulated genes, pro-inflammatory cytokines, chemokines, and transcription factors genes implicated in the immune response. These findings are in agreement with a recent study by Bengue and colleagues, which demonstrates that MAYV is also capable of infecting human brain cells and eliciting a strong antiviral response [49]. Additional research groups have demonstrated that other arboviruses, including chikungunya virus, West Nile virus and Zika virus, also efficiently infect HDFs and similarly induce an antiviral state in these cells [50,51].

Having demonstrated that HDFs are a feasible model for MAYV infection, we decided to study the effect of the MAPK inhibitors SB203580, SP600125, and U0126 on MAYV replication. Our results indicate that the p38 inhibitor SB203580 promotes a significant dose-dependent reduction in MAYV titers in both HDFs and HeLa cells. On the other hand, the inhibition of the JNK or ERK1/2 pathways with SP600125 or U0126, respectively, only appears to slightly affect MAYV replication in HeLa cells. Importantly, the observed effects of MAPK inhibitors on MAYV replication did not appear to result from virucidal activity by these compounds. Previous reports have shown that p38 inhibition affects the replication of viruses from different families, among them: enterovirus 71, hepatitis C virus, influenza virus, chikungunya virus, and SARS-CoV-2 [27,35,52,53,54].

In an attempt to explore a possible mechanism by which MAPK inhibitors disrupt MAYV replication, we analyzed the expression of structural E1 protein in HDFs or HeLa cells treated with these compounds. Our data reveal that the p38 inhibitor SB203580 results in a robust attenuation of viral E1 protein expression in both the cell lines tested. Conversely, we only observed a slight reduction in viral E1 protein expression following inhibition of the JNK or ERK1/2 pathways, suggesting again that p38 may have an important function in MAYV replication. These findings are in agreement with prior studies which show that blocking p38 activity can reduce the expression of several viral proteins [27,53,55].

In order to further prove p38′s importance in the MAYV replication process, we performed a p38α and p38β isoform knockdown experiment in HDFs infected with MAYV using a new class of molecules known as PROTRACs. PROTACs technology allows any protein to be targeted for degradation through an ubiquitin-proteasome system-dependent mechanism [38]. Recently, Donoghue and collaborators demonstrated that the PROTACs compound NR-7h is able to induce p38α and p38β isoform degradation, thus blocking downstream p38 signaling [41]. Similarly, our data reveal that knockdown of the p38α and p38β isoforms in NR-7h-treated HDFs promotes a dose-dependent decline in MAYV antigen-positive cells and viral titers. Moreover, we observed a decrease in viral E1 protein expression, confirming that p38 could play an important role in MAYV replication. Previous work has shown that PROTAC compounds, which directly target several viral proteins, may be an antiviral therapeutic strategy [56,57].

Given the prominent role of p38 kinase in controlling a plethora of cellular processes, its dysregulation has been associated with numerous pathologies [40]. Thus, the pharmaceutical industry has developed diverse inhibitory molecules targeting this kinase [39]. Among them, Losmapimod is a potent p38α and p38β isoform inhibitor that has been evaluated in different clinical trials and shown to be well-tolerated and safe [44,45]. In order to validate our previous findings, we treated HDFs with Losmapidmod and then evaluated the impact on MAYV replication. Our results indicate again that p38 inhibition with this drug affects MAYV replication in a dose-dependent manner. In addition, we observed a reduction in viral E1 protein expression, suggesting that p38 might serve as a therapeutical target to control MAYV infection. Zhang and collaborators showed that Losmapimod is able to inhibit Lassa virus entry, although through a mechanism not involving p38 inhibition [58]. In another study, Losmapimod was shown to disrupt SARS-CoV-2 replication in a model of primary alveolar epithelial cells, suggesting that p38 is a potential target for COVID-19 treatment [59]. In fact, Losmapimod is currently being tested in a clinical trial with COVID-19-infected patients [40].

Though our in vitro analyses point to p38 kinase as a probable cellular target to treat MAYV infection, these findings should be corroborated in exhaustive animal studies to determine the feasibility of using p38 inhibitors as antiviral drugs. Nevertheless, based on our results, we propose that p38 kinase plays a key role in MAYV replication and highlight this MAPK as a therapeutical target candidate to combat MAYV infection.

## Data Availability

Not applicable.

## Supplementary Materials

The following are available online at https://www.mdpi.com/article/10.3390/v13061156/s1, Table S1: List of primers used in this study. Figure S1: MAYV induces a strong time-dependent cytopathic effect in primary HDFs. Figure S2: MAPK inhibitors SB203580, SP600125 and U0126 show no virucidal activity against MAYV. Figure S3: Knockdown of p38α and p38β isoforms using the PROTAC compound NR-7h in HDFs.
