# Supplementary material for: Inhibition of p38 Mitogen-Activated Protein Kinase Impairs Mayaro Virus Replication in Human Dermal Fibroblasts and HeLa Cells
Source: Viruses. 2021 Jun 17;13(6):1156. doi: 10.3390/v13061156 (PMC8233896; doi:10.3390/v13061156)
Supplement: Supplementary file 1 [file viruses-13-01156-s001.zip › viruses-1215146-SI.pdf]

viruses

Supplementary material

Inhibition of p38 Mitogen-Activated Protein Kinase Impairs Mayaro Virus Replication in Primary Human Dermal Fibroblasts and HeLa Cells

Madelaine Sugasti [1,2], Yessica Y. Llamas-González [1,3], Dalkiria Campos [1] and José González-Santamaría [1,*]

[1] Grupo de Biología Celular y Molecular de Arbovirus, Instituto Conmemorativo Gorgas de Estudios de la Salud, Panamá 0816-02593, Panamá; msugasti@gorgas.gob.pa (M.S.); qfb.y.llamas@gmail.com (Y.Y.L.G.); dcampos@gorgas.gob.pa (D.C.)
[2] Programa de Maestría en Microbiología Ambiental, Universidad de Panamá, Panamá, 3366, Panamá;
[3] Programa de Doctorado en Ciencias Biológicas, Universidad de la República, Montevideo 11200, Uruguay
* Correspondence: jgonzalezsantamaria@gorgas.gob.pa; Tel.: +507-527-4814

Table S1. List of primers used in this study.

Gene	Primer sequences (5′-3′)	References
MDA5	Forward: GCCATTGCAGATGCAACCAG<br>Reverse: TTGCGATTTCCTTCTTTTGCAG	[1]
ISG15	Forward: GAGAGGCAGCGAACTCATCT<br>Reverse: CTTCAGCTCTGACACCGACA	[2]
MxA	Forward: GGTGGTGGTCCCCAGTAATG<br>Reverse: ACCACGTCCACAACCTTGTCT	[3]
RIG-I	Forward: TGTTCTCAGATCCCTTGGATG<br>Reverse: CACTGCTCACCAGATTGCAT	[4]
OAS2	Forward: AAACCAGGCCTGTGATCTTG<br>Reverse: GGGCTATTTCCAGACAACGC	[1]
AIM2	Forward: ATCAGGAGGCTGATCCCAAAG<br>Reverse: TCTTCATCACTGCAGACACCG	[1]
IL1-β	Forward: AACCTCTTCGAGGCACAAGG<br>Reverse: GTCCTGGAAGGAGCACTTCAT	[1]
TNF-α	Forward: CAGAGGGAAGAGTTCCCCAGGGACC<br>Reverse: CCTTGGTCTGGTAGGAGACGG	[5]
IL-6	Forward: TGTGAAAGCAGCAAAGAGGCACTG<br>Reverse: ACAGCTCTGGCTTGTTCCTCACTA	[4]
RANTES	Forward: GAGGCTTCCCCTCACTATCC<br>Reverse: CTCAAGTGATCCACCCACCT	[4]
IL-8	Forward: CAGCCAAAACTCCACAGTCA<br>Reverse: TTGGAGAGCACATAAAAACATCT	[4]
TLR3	Forward: TGGGACCAAGGCAAAGGAGT<br>Reverse: TTCTCTTGGTTGGGCCACCT	[1]
TLR7	Forward: AATGTCACAGCCGTCCCTAC<br>Reverse: TTATTTTTACACGGCGCACA	[1]
IRF3	Forward: GATGCACAGCAGGAGGATTT<br>Reverse: GATTTTATGTGGGTCGTGAGG	[1]
IRF7	Forward: CAGAGTCTTCTTCCAAGAGCTG<br>Reverse: TGCTATCCAGGGAAGACACA	[1]
IFN-*α*	Forward: GCCTCGCCCTTTGCTTTACT<br>Reverse: CTGTGGGTCTCAGGGAGATCA	[5]
IFN-β	Forward: ATGACCAACAAGTGTCTCCTCC<br>Reverse: GCTCATGGAAAGAGCTGTAGTG	[5]
β-actin	Forward: AGAGCTACGAGCTGCCTGAC<br>Reverse: AGCACTGTGTTGGCGTACAG	[6]

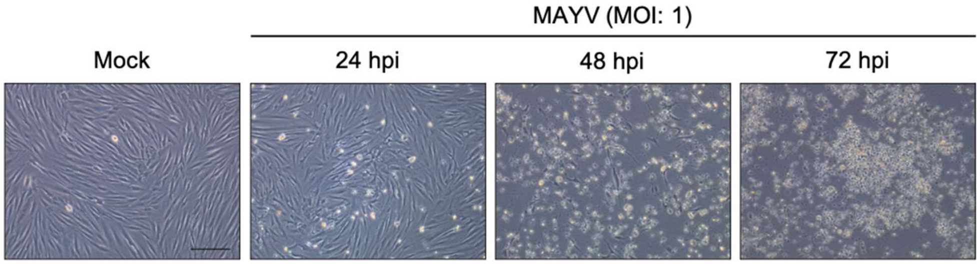

Figure S1. Mayaro virus induces a strong, time-dependent cytopathic effect in primary HDFs. HDFs were infected with MAYV at an MOI of 1. At indicated times after infection, images were captured using an inverted microscope and a MCI70-HD camera (Leica). Scale bar: 100 μm.

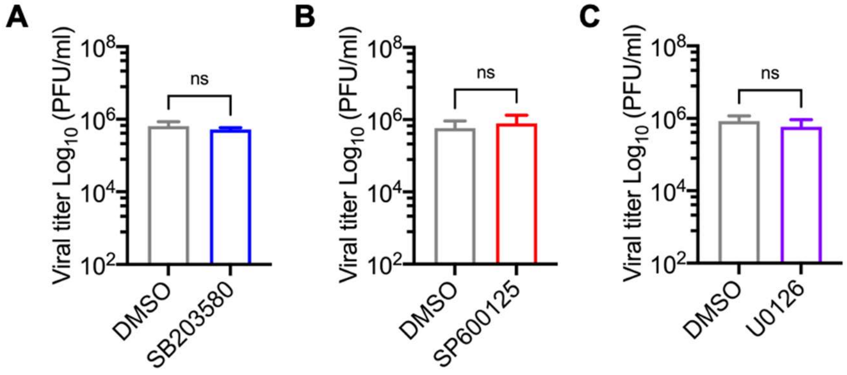

Figure S2. MAPK inhibitors SB203580, SP600125 and U0126 show no virucidal activity against MAYV. 1 x 10^6 PFU of MAYV in serum-free MEM were incubated with SB203580 (A), SP600125 (B) or U0126 (C) at 37 °C for 1 h. Then, the remaining virus for each experimental condition was directly quantified using plaque assay. Statistical differences were analyzed using the Mann-Whitney test: ns: non-significant.

viruses

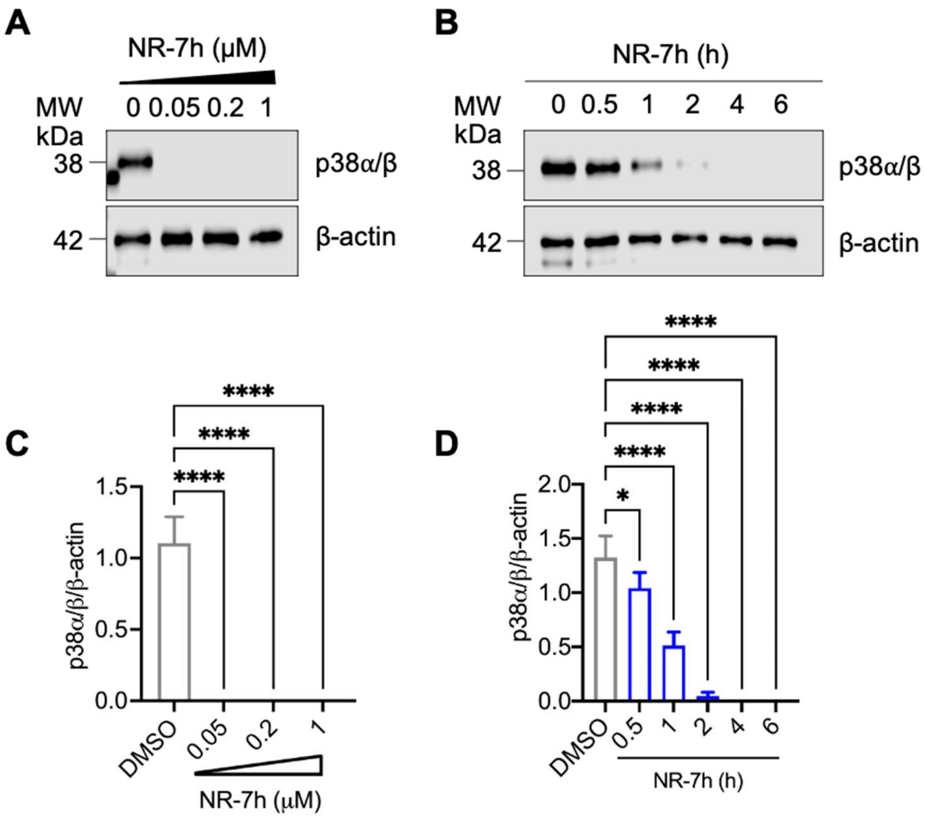

Figure S3. Knockdown of p38α and p38β isoforms using the PROTAC compound NR-7h in HDFs. (**A**) HDFs were treated with increasing concentrations of NR-7h; after 24 h of incubation, p38α/p38β protein levels were analyzed using immunoblot. β-actin protein was used as a loading control. MW: molecular weight. kDa: kilodaltons. (**B**) HDFs were treated with NR-7h at a concentration of 1 μM; at different time points, p38α/p38β protein levels were evaluated as described above. (**C**, **D**) Densitometric analysis of p38α/p38β proteins was performed using ImageJ software and normalized with β-actin protein. Statistical differences were analyzed using the One-way ANOVA test followed by Dunnett's post-test: *$p < 0.05$ and ****$p < 0.0001$.

References

1. Hamel, R., et al., *Biology of Zika Virus Infection in Human Skin Cells.* J Virol, 2015. **89**(17): p. 8880-96.
2. Bektas, N., et al., *The ubiquitin-like molecule interferon-stimulated gene 15 (ISG15) is a potential prognostic marker in human breast cancer.* Breast Cancer Res, 2008. **10**(4): p. R58.
3. Stevenson, N.J., et al., *Ribavirin enhances IFN-alpha signalling and MxA expression: a novel immune modulation mechanism during treatment of HCV.* PLoS One, 2011. **6**(11): p. e27866.
4. da Conceicao, T.M., et al., *Essential role of RIG-I in the activation of endothelial cells by dengue virus.* Virology, 2013. **435**(2): p. 281-92.
5. Devhare, P.B., et al., *Analysis of antiviral response in human epithelial cells infected with hepatitis E virus.* PLoS One, 2013. **8**(5): p. e63793.
6. Chen, F., et al., *High-efficiency generation of induced pluripotent mesenchymal stem cells from human dermal fibroblasts using recombinant proteins.* Stem Cell Res Ther, 2016. **7**(1): p. 99.
